# Research into the Disintegration of Abrasive Materials in the Abrasive Water Jet Machining Process

**DOI:** 10.3390/ma14143940

**Published:** 2021-07-14

**Authors:** Andrzej Perec

**Affiliations:** Faculty of Technology, Jacob of Paradies University, 66-400 Gorzow Wielkopolski, Poland; aperec@ajp.edu.pl

**Keywords:** abrasive materials, garnet, corundum, olivine, disintegration, cutting process efficiency

## Abstract

The size and distribution of abrasive particles have a significant influence on the effectiveness of the cutting process by the high-speed abrasive water jet (AWJ). This paper deals with the disintegration intensity of abrasive materials in AWJ cutting during the creation of the abrasive jet. An evaluation of the abrasive materials was performed after forming in the cutting head was carried out and grain distribution was evaluated using the geometric and logarithmic Folk and Ward method. The influence of the abrasive concentration of abrasive materials such as alluvial garnet, recycled garnet, corundum, and olivine on grain distribution was studied. A recovery analysis was also carried out and the recycling coefficient was determined for each abrasive material tested.

## 1. Introduction

The size and shape of abrasive grains [1,2] are important in abrasive machining processes for achievement of both high efficiency and high accuracy [3,4,5] of the treated surface. During the injection of the abrasive into the jet within the mixing chamber and the focusing tube of the cutting head, the intensive abrasive material disintegrates [6], which is one of the characteristic properties of this process [7]. Because abrasive grain size distribution is essential to the effectiveness of most abrasive cutting processes [8,9], especially in abrasive water jet (AWJ) [10], it seems advisable to carry out detailed research into this aspect in order to achieve optimum efficiency.

The main focus and originality of the research presented is to show the influence of abrasive concentration in the AWJ on its disintegration. For this purpose, the most popular abrasive was selected—GMA80 alluvial garnet. This is the basis for comparison with the other abrasives tested: recycled garnet, corundum, and olivine. This study is an extension of the preliminary results published earlier in a much smaller scope [11].

**Figure 1:**
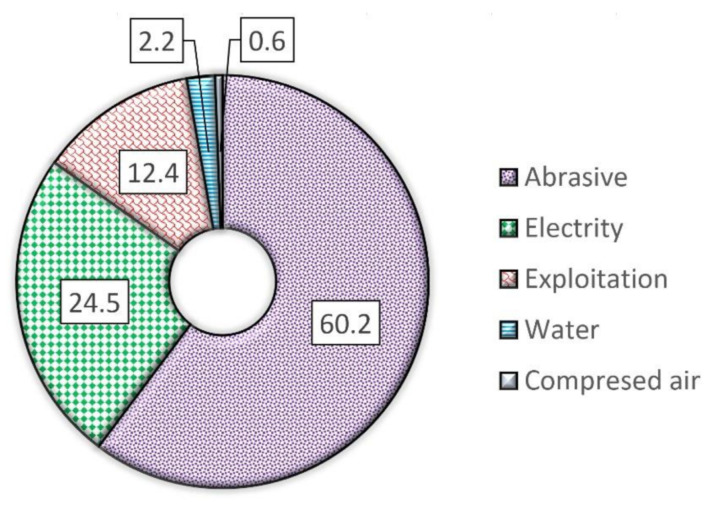
Example percentage cutting costs for the Omax 55100 machining center without amortization costs. Media use: garnet abrasive 24 kg/h, electrical energy 30 kWh, water 270 dm^3^/h, compressed air 300 dm^3^/h [12].

The other factor in favor of undertaking such research is cost effectiveness. The cost of most garnet abrasive materials is up to 60% of the total cost of AWJ technology [12], which results in its limited use in materials cutting operations. The high cost of garnet abrasives when compared with other constituents (Figure 1) is a result of the relatively high price of garnets from India, Australia, and the USA.

**Figure 2:**
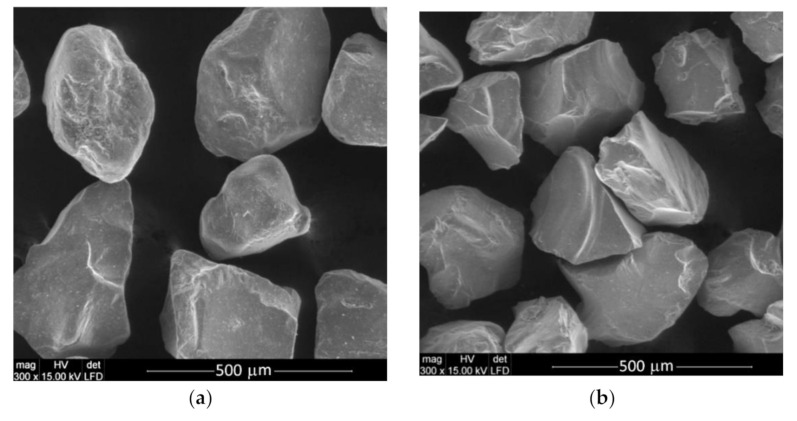
Exemplary scanning electron microscope (SEM) view of garnet abrasive material: (**a**) fresh, before AWJ machining process; (**b**) recycled after AWJ machining process. Magnification 300×.

Another way to reduce machining costs is by recycling the used abrasive. Among the abrasives, especially alluvial garnet, the recovered abrasive turns out to be more effective as the cutting edges are sharper (Figure 2). Additionally, recycling of the abrasive materials can mean the abrasive water jet technique results in more savings, is effective, and environmentally friendly [13]. For these reasons, research on the recycling of abrasives, especially garnet, is the subject of research in various scientific centers.

Chetty et al. [14] published the results of regional garnet abrasives recycling in the aluminum cutting process by means of an abrasive water jet. The impact of pressure, traverse speed, and abrasive flow rate has an effect on grain size, depth of cut, width, and the taper of the cut kerf. Recycled abrasives reduce kerf taper and improve the parallelism of the cut surface. These effects show that the appropriate selection of abrasive grain size distribution is essential to achieve better results.

The determination of the extent of changes in the size of particles induced in the cutting head have been published by Hlavac et al. [15]. They recognized that information about the grain size at the cutting head output is essential for examining further processes, e.g., the impingement of the water stream on industrial mineral particles on a solid-state object or the impact of such water stream on the fluid barrier or reverse flow of the same type.

Aydin [16] presented a study in which the effect of rock properties affecting the recycling of abrasives in AWJ cutting was investigated. An abrasive mass percentage above 106 μm was considered as a performance criterion in terms of recycling of abrasives since these abrasives can be effectively reused in the rock cutting applications. The study revealed that a substantial quantity of the used abrasive particles are in a reusable condition.

The cutting efficiency of multiple recycling garnet abrasive materials [17] was also tested for their effects on cutting depth, kerf width, kerf taper angle, and surface roughness. After each pass, the retrieved abrasive particles were dried and sieved to determine their disintegration behaviors. Garnet grains smaller than 106 μm were rejected. A substantial number of used abrasives qualified for reuse in the cutting. The recycling factor of abrasives were determined to be over 81% after the first pass to over 17% after the fourth pass.

Gye-Chun Cho et al. [18] carried out rock cutting research with different sizes of garnet grain to find the influence of its flow rate, physical characteristic, and grain size distribution on rock cutting efficiency. Additionally, garnet grain disintegration was tested for the abrasive water jet. The test results demonstrated that the grain size distribution, garnet purity, density, and hardness are the most important factors for rock cutting efficiency. The research presented a better understanding of the effectiveness of coarse garnet with respect to the characteristics of the garnet.

A feasibility study of abrasive materials usage under a 400 MPa AWJ was presented by Perec [11]. Garnet and fused alumina were tested and preliminary results on the disintegration of these abrasive materials were shown. Of major importance is the increase in cutting capacity with crushed grains due to the formation of new cutting edges on the grains. 

Under study, disintegration showed differences in results on machining efficiency and presented wear of the focusing tube—the key element in formatting the jet. Additionally, the work aimed at reducing the size of the abrasive grains and conducting research on using a water jet for disintegration of mineral materials [19,20,21]. The impact of the internal shape of the mixing chamber on the particle comminution in the water stream process was researched experimentally with a connection to the reverse jet disintegration of materials [22].

## 2. Materials and Methods

Abrasive grains utilized in AWJ cutting systems have one of the most significant influences on cutting kerf properties. The most commonly used abrasive material is mineral garnet. Its wide use in high-pressure water jets as the abrasive material is a result of being able to attain high efficiency at comparatively low cost to focusing tube durability [23]. Alternative natural and synthetic abrasives, such as aluminum oxide [24] or olivine [25], may also be used. In the case of cutting with an abrasive suspension water jet (ASWJ), quartz sand can be used as abrasive material [26]. To achieve a compromise between focusing nozzle life and the efficiency of machining, careful selection of abrasive material is endorsed [27].

### 2.1. Alluvial Garnet

The Australian garnet used in the research is mined from an alluvial deposit near Geraldton in Western Australia by the GMA Garnet company (Perth, WA, Australia). The Australian garnet has an extremely low dust level and advanced processing methods guarantee a low chloride content. Rinsing with spring water is used to reduce the quantity of soluble salts to less than 10 ppm, and roasting is used to burn off any organic matter. Double screening is used to ensure an adequate grain distribution. Its detailed properties are presented in Table 1.

### 2.2. Recycled Garnet

This abrasive is a recycled product of alluvial garnet, manufactured by GMA Garnet (Middle East) FZE in Jebel Ali Dubai, United Arab Emirates. The GMA Garnet abrasive can be recycled up to five times without compromising performance [29]. Some increase in cutting efficiency is possible when using recycled abrasive, primarily due to good abrasion resistance and low brittleness. Recycling provides garnet users with a cost-effective and environmentally sound opportunity to dispose of used abrasive that would otherwise be disposed of as industrial waste.

### 2.3. Corundum

Corundum is a crystalline form of aluminum oxide (Al_2_O_3_). Among natural abrasives, only diamond is harder. It does not react with acids and most basic environments. Deposits of corundum occur in contact with metamorphic rocks (emery) and gemstones (sapphire and ruby). The most popular forms of corundum are translucent corundum and emerald corundum. It is defined by high level of hardness, good strength, and shape. Tools made of this abrasive material are suitable for grinding metals with high tensile strength, such as: carbon steel, general-purpose alloy steel, annealed malleable cast iron, and hard melt, etc. It can also be used as a refractory material. It is also widely used in the constructional production of coated abrasive tools, especially for metal grinding.

The corundum used in the research (fused alumina) was produced by Dengfeng Sweet Abrasives Co., Ltd. based in Dengfeng City, China. Its properties are presented in Table 2.

### 2.4. Olivine

Olivine is in fact the name of an isomorphic series of minerals lying between two extreme elements: fayalite and forsterite. Fayalite is a pure iron compound of Fe_2_SiO_4_, while forsterite is a pure magnesium compound of Mg_2_SiO_4_.

Green Lightning olivine, produced by SIBELCO NORDIC AS in Rud, Norway, from a deposit on the west coast of Norway, south of Ålesund, was used in the research. Green Lightning olivine is a magnesium iron silicate with the highest magnesium content. Green Lightning is made from “Dunite rock” and does not contain free silica. Properties of olivine used in the research are presented in Table 3.

### 2.5. Apparatus and Method

The main devices used in the test were a Intensifier type I50 byKMT Waterjet Systems Inc., Baxter Springs, KS, USA and CNC table type ILS55 by TECHNI Waterjet, Campbellfield, Victoria, Australia) with a dedicated PC control system.

To collect abrasive material after discharge from the cutting head, a unique catcher was designed and used. This receiver performs the function of collecting the material and preventing further disintegration of the abrasive materials after exiting the cutting head. The bottom PVC catcher was shielded by a hard metal plate to prevent its perforation.

Research was conducted under pressure of 390 MPa at 0.25 mm ID water nozzle and 0.76 mm ID focusing tube. The variable control parameter was the concentration of abrasive in the jet. The following values were adopted for the tests: 15, 17.5, 20, 22.5, and 25%.

For evaluation of the grain distribution of the abrasive materials, Retsch screening equipment was utilized. The mass of abrasive materials left on the screens was weighed on the digital lab scales. The statistics of the abrasive materials grains distribution were calculated using the Gradistat v.9.1 program by Kenneth Pye Associates Ltd., Wokingham, UK [32] based on the Folk and Ward method [33].

Observation of selected abrasive grains was performed using an Quanta 200 FEG MKII scanning electron microscope by FEI Technologies Inc., Hillsboro, OR, USA under high vacuum conditions and a 15 kV accelerating voltage. 

## 3. Results and Discussion

### 3.1. Abrasive Grain Disintegration

#### 3.1.1. Alluvial Garnet

**Figure 3:**
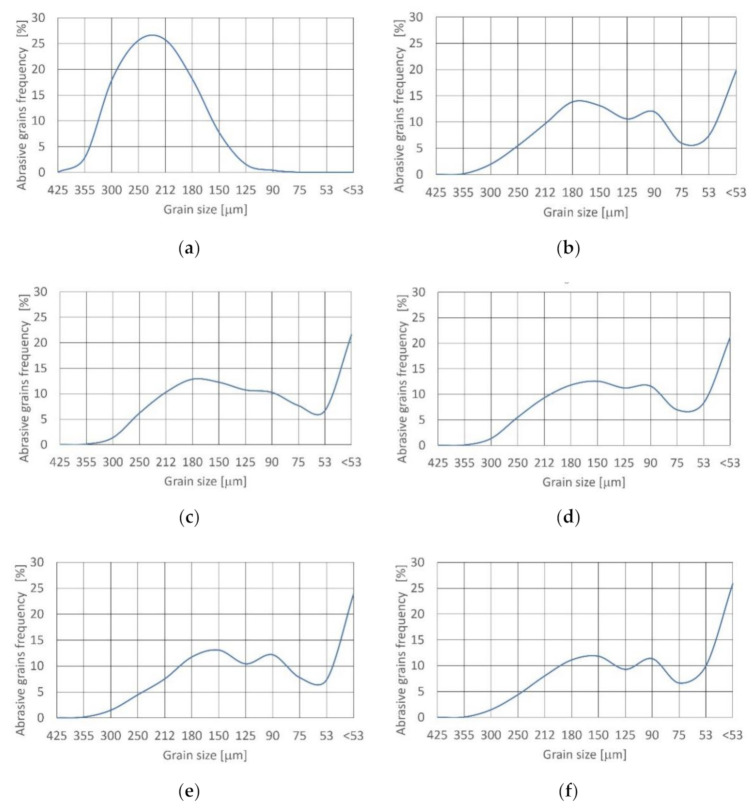
Size distribution of alluvial garnet grain at concentration: (**a**) fresh; (**b**) 15%;(**c**) 17.5 %;(**d**) 20%;(**e**) 22.5%; (**f**) 25%.

The impacts of the alluvial garnet abrasive disintegration in the cutting head during jet formation are presented in Figure 3. The distribution of fresh grains is unimodal and symmetrical (Table 4). Skewness is near zero. Distribution is mesokurtic (0.9 < K_G_ < 1.11).

After leaving the cutting head at 15% concentration, density function of distribution shifted to bimodal and asymmetrical (very fine skewed) with minus asymmetry. Skewness equaled −0.304. Kurtosis is 0.67 < K_G_ < 0.9 and distribution is platykurtic.

Increasing the concentration value to 17.5% results in a change of density function of distribution to the trimodal and asymmetrical (fine skewed), with negative asymmetry. Skewness is equal to −0.285. Distribution is also platykurtic as result of kurtosis (0.67 < K_G_ < 0.9).

Further increasing the concentration to 20% maintains distribution and skewness. Increasing the concentration over 22.5% leads to the achievement of very platykurtic distribution, because kurtosis reaches K_G_ < 0.67. The density function of distribution remains unchanged (trimodal and asymmetrical, fine skewed), with minus asymmetry. 

For all concentration values the frequency of grains from 425 to 250 μm reduced drastically and the superiority of the grains below 53 μm appeared. This had not previously been noted [34].

#### 3.1.2. Recycled Garnet

**Figure 4:**
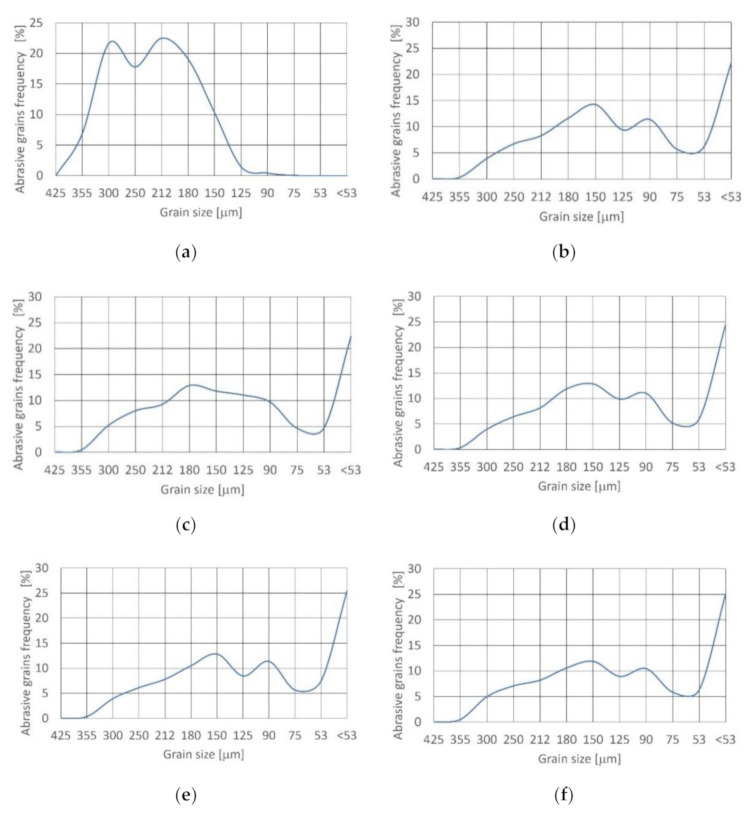
Size distribution of recycled garnet grain at concentration: (**a**) fresh; (**b**) 15%; (**c**) 17.5%;(**d**) 20%; (**e**) 22.5% and (**f**) 25%.

The outcomes of the recycled garnet abrasive disintegration tests in the cutting head during jet formation are presented in Figure 4. The density function approximating the distribution of fresh grains (Table 5) is bimodal and symmetrical (skewness is near zero). The distribution is platykurtic because kurtosis = 0.67 < K_G_ < 0.9.

After passing through the cutting head at 15 and 17.5% concentration, the density function of distribution is still bimodal and asymmetric (fine skewed), with negative asymmetry. Skewness is equal to −0.272 to −0.298 and the distribution is platykurtic (kurtosis = 0.67 < K_G_ < 0.9).

Increasing the concentration value over 20% results in a change of distribution to very platykurtic because kurtosis K_G_ < 0.67. The density function of distribution is maintained at bimodal and asymmetric (fine skewed), with negative asymmetry. Skewness is −0.285.

Further increasing the concentration to 25% results in a change of density function of distribution to the trimodal and asymmetric (fine skewed), with negative asymmetry. Skewness is equal to −0.206. Distribution is very platykurtic as a result of kurtosis at K_G_ < 0.67.

For each concentration value the frequency of grains from 425 to 250 μm decreased radically and the dominance of the grains below 53 μm was noted for the first time.

#### 3.1.3. Corundum

**Figure 5:**
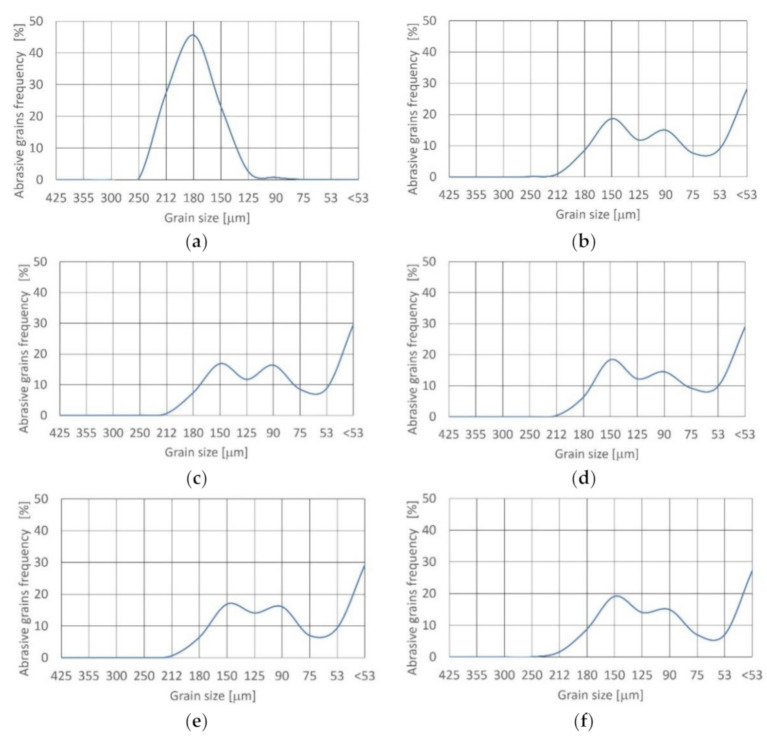
Size distribution of corundum grain at concentration: (**a**) fresh; (**b**) 15%; (**c**) 17.5%; (**d**) 20%; (**e**) 22.5%; and (**f**) 25%.

The effects of corundum abrasive disintegration in the cutting head through jet formation are presented in Figure 5. The density function approaching the distribution of fresh grains is unimodal and symmetrical (Table 6) since skewness is near zero. Distribution is mesokurtic (0.9 < K_G_ < 1.11).

After passing through the cutting head at 15% concentration, the density function of distribution changes to bimodal and asymmetric (fine skewed), with negative asymmetry. Skewness is equal to −0.162 and distribution is very platykurtic (kurtosis K_G_ < 0.67).

Increasing the concentration value to 20% results in a change of density function of distribution to the trimodal and symmetrical. Skewness is equal to −0.094. Distribution is also very platykurtic (kurtosis K_G_ < 0.67). Further increasing the concentration causes a return to bimodal and asymmetric (fine skewed), with negative asymmetry. Distribution remains very platykurtic (kurtosis K_G_ < 0.67).

For all concentrations of abrasive grains in the jet, the frequency of grains from 212 to 180 μm decreased significantly and the dominance of grains below 53 μm increased, which was noted for the first time.

#### 3.1.4. Olivine

**Figure 6:**
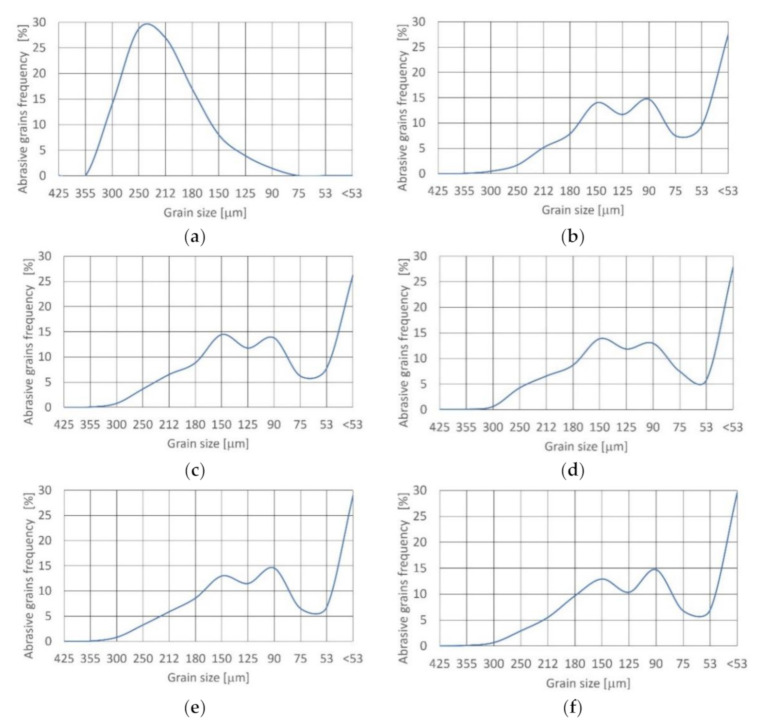
Size distribution of olivine grain at concentration: (**a**) fresh; (**b**) 15%; (**c**) 17.5%; (**d**) 20%; (**e**) 22.5% and (**f**) 25%.

The effects of the study of olivine abrasive disintegration in the cutting head at the period of jet formation is shown in Figure 6. The density function approximating the distribution of fresh grains is unimodal and fine skewed (Table 7) because skewness is −0.137. Distribution is mesokurtic (0.9 < K_G_ < 1.11).

After passing through the cutting head at 15% concentration, density function of distribution shifts to bimodal and asymmetric (fine skewed), with negative asymmetry. Skewness is equal to −0.104. Distribution is very platykurtic (kurtosis K_G_ < 0.67).

For the 17.5% concentration, the density function of distribution is still bimodal and asymmetric (fine skewed), with negative asymmetry. Skewness is equal to −0.272 to −0.298 and distribution is again very platykurtic.

After passing through the cutting head at 20% concentration, the density function of distribution shifts to trimodal. Asymmetric (fine skewed) character with negative asymmetry remains unchanged. Skewness is equal to −0.169. Distribution is very platykurtic (kurtosis K_G_ < 0.67).

Further increasing the concentration to 22.5% and over results in a reversion to a bimodal density function of distribution and leads to retention of very platykurtic distribution (kurtosis K_G_ < 0.67) and fine skewness with negative asymmetry.

For all concentrations of abrasive in the jet, the frequency of grains from 300 to 212 μm decreased significantly and a predominance of grains below 53 μm appeared for the first time.

### 3.2. Comparison of Abrasive Grain Distribution Changes

The Folk and Ward graphic and logarythmic methods were used to compare the distribution of abrasive after disintegration in the cutting head. This approach is rather insensible to samples including the wide scope of particle sizes in the tails of the distribution [32]. Therefore, the Folk and Ward methods provide the most solid basis for practical comparisons of specimens of varying composition [33].

**Figure 7:**
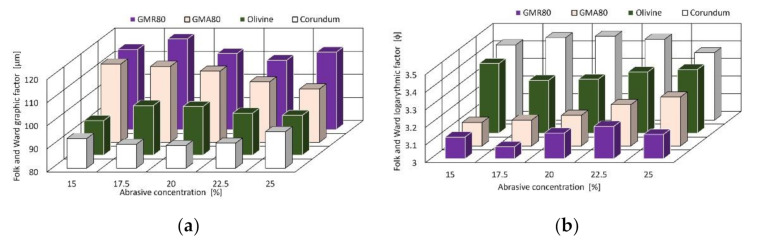
Folk and Ward sample means distribution factors: (**a**) graphic; (**b**) logarithmic.

Figure 7 shows a sample of traditionally logarithmic statistics (in phi units), and expressed geometrically (in micrometers units) for means.

In both cases, it can be observed that the change in concentration in the tested range for each abrasive type has relatively little effect on the value of the Folk and Ward coefficients. The difference for the mean geometric coefficient does not exceed 10%, and for the logarithmic mean coefficient, it is less than 4%. The other statistical measures of distribution, namely, sorting, skewness, and kurtosis, are also similar for all tested abrasives (Table 4, Table 5, Table 6 and Table 7). This shows that the particle size distributions are also similar for each abrasive in the whole tested range. This is confirmed in the particle size distribution charts presented above.

### 3.3. Recycling of Abrasive Materials

**Figure 8:**
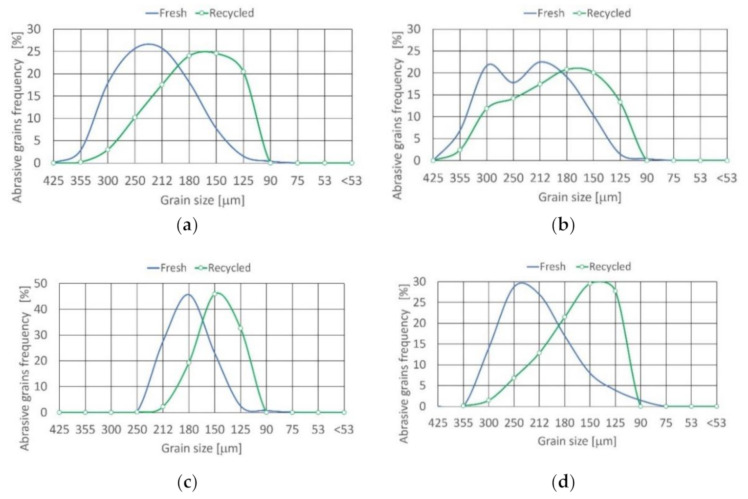
Size distribution of recycled abrasive materials: (**a**) alluvial garnet, (**b**) recycled garnet, (**c**) corundum, (**d**) olivine.

After obtaining the abrasive and drying it, sieve analysis was executed, and the mass of each fraction was calculated. For all of the materials tested, abrasive size range is within the range of 350 to 125 μm. To establish what fraction of the original range is found in the jet formed in the cutting head, all of the fractions smaller than the lower limit of the particle distribution were rejected as ineffective in the cutting process [12]. This is illustrated in Figure 8. Details of abrasive material distribution are presented in Table 8.

In the recycling process of alluvial garnet, grains smaller than the limit (for grains #80 it is 90 μm) was removed as being inefficient during the cutting process. A very small positive asymmetry density function approximating the grain distribution is visible. Skewness is equal to 0.071 and distribution is mesokurtic (0.9 < K_G_ < 1.11). 

In the case of recycled garnet, similar to alluvial garnet, grains smaller than the 90 μm were removed as ineffective in the cutting operations. The density function approaching the distribution is unimodal and almost symmetrical because skewness is close to zero. Kurtosis is in the range of 0.67 <K_G_ < 0.9; here, the distribution is platykurtic.

For corundum recycled grains, the density function describing the distribution is unimodal and practically symmetrical (skewness is equal to 0.029). Distribution is mesokurtic (0.9 < K_G_ < 1.11).

In the recycling of olivine grains, those smaller than the limit (for grains #60 it is 125° μm) were removed as being unproductive in the cutting. The asymmetry (coarse skewed) density function approximating the recycled grain distribution is also visible.

The recycling rate was calculated on the basis of Equation (1):

*C*_r_ = *m*_r_/*m*_t_ (1)

where:

*C*_r_ is the abrasive recycling coefficient [g/g]

*m*_r_ is the mass of recycled abrasive [g]

*m*_t_ is the total mass of abrasive [g] 

**Figure 9:**
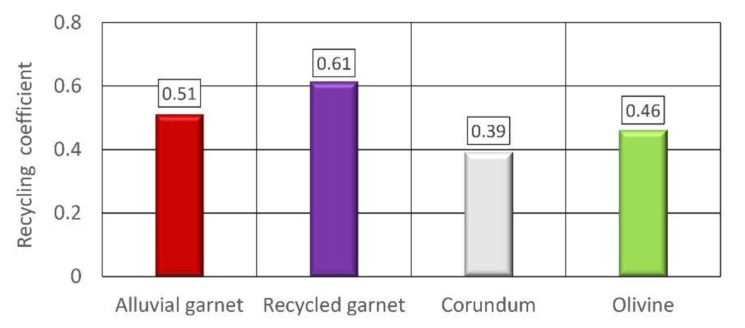
Recycled coefficient for tested abrasive materials.

Specific results for the tested abrasives are presented in Figure 9. A recycling coefficient, amounting to 0.61 was observed for the recycled garnet. 

The second biggest value was noted for alluvial garnet. This implies it can be estimated that it is possible to reuse over half of the expended abrasive.

The following abrasives were olivine and corundum with the recycling coefficients of 0.46 and 0.39, respectively. This means that over one-third of the abrasive can be used a second time.

In the case of corundum, apart from having the lowest recycling rate, a major obstacle to its widespread use for AWJ cutting is the intensive wear caused to the focusing tube [24].

## 4. Conclusions

Based on this research, in relation to the previously published preliminary studies of grains refinement during jet formation in the cutting head [18], even more effects of disintegration were observed in testing.

For all tested abrasives, intense size reduction of abrasive grains was observed during the jet formation in the cutting head. In this normally considered, unfavorable phenomenon of disintegration of the abrasive grains, it has been observed that the disintegrated abrasive grains reveal new, sharp corners and cutting edges, which is beneficial because they can be used to increase the efficiency of the cutting process.

It deserves special mention that it was observed that the effect of abrasive concentration in the stream in the entire tested range from 15 to 25% has no significant effect on the degree and nature of the abrasive grains disintegration.

For garnet abrasives disintegrated at all concentration values, a predominance of 20–25% appeared on the grains below 53 μm. For all other abrasives that disintegrated with each value of concentration values, the predominance of grains below 53 μm is higher and equals 30%.

Based on the research, the following detailed conclusions were drawn:

- The biggest recycling potential, from 51–61% range, characterized garnet;
- For olivine, the recycling potential is equal to 46%;
- The recycling potential of corundum is almost 40%, but abrasive wear of the focusing tube is much greater than for garnet.

An additional benefit is the possibility of reusing the abrasive materials as a full-fledged product. This can help to resolve the problem of the accumulation of used abrasives generated by waterjet cutting in landfills, on a massive scale. 

In the future work, the effects of shape-changing of abrasive grains formed in the AWJ cutting head will be tested.

## Figures and Tables

**Table 1:** Detailed properties of Australian alluvial garnet [28].

Properties	Units	Value
Chemical composition		
Fe_3_Al_2_(SiO_4_)_3_	%	>97.0
FeTiO_3_	%	<2.0
CaCO_3_	%	<1.00
ZrSiO_4_	%	<0.10
SiO_2_	%	<0.10
Melting point	°C	1250
Density	kg/m^3^	4100
Bulk Density	kg/m^3^	2300
Hardness	Mohs	7.5–8.0

**Table 2:** Detailed properties of corundum [30].

Properties	Units	Value
Chemical composition		
Al_2_O_3_	%	94.5–95.5
SiO_2_	%	1.33–1.50
Fe_2_O_3_	%	0.18–0.30
Ti_2_O	%	2.45–3.50
CaO	%	0.11–0.30
Melting point	°C	2050
Density	kg/m^3^	3900
Hardness	Mohs	9.0

**Table 3:** Detailed properties of Norwegian olivine [31].

Properties	Units	Value
Chemical composition		
MgO	%	48.8–49.7
SiO_2_	%	41.5–41.9
Fe_2_O_3_	%	7.3–7.6
Al_2_O_3_	%	0.4–0.5
Cr_2_O_3_	%	0.31–0.66
Density	kg/m^3^	3300
Bulk Density	kg/m^3^	1700
Hardness	Mohs	6.5–7.0

**Table 4:** Alluvial garnet grains distribution statistics.

Concentration (%)		Fresh	15	17.5	20	22.5	25
Folk and Ward Method (μm)	MEAN	244.6	113.9	112.9	110.8	106.2	103.1
	SORTING	1.273	1.907	1.920	1.901	1.891	1.899
	SKEWNESS	−0.039	−0.304	−0.285	−0.259	−0.212	−0.121
	KURTOSIS	0.920	0.719	0.676	0.678	0.634	0.604
Folk and Ward Method (φ)	MEAN	2.032	3.134	3.147	3.174	3.235	3.278
	SORTING	0.348	0.931	0.941	0.927	0.919	0.925
	SKEWNESS	0.039	0.304	0.285	0.259	0.212	0.121
	KURTOSIS	0.920	0.719	0.676	0.678	0.634	0.604

**Table 5:** Recycled garnet grains distribution statistics.

Concentration (%)		Fresh	15	17.5	20	22.5	25
Folk and Ward Method (μm)	MEAN	246.4	114.7	119.2	112.9	110.0	113.5
	SORTING	1.315	1.964	2.007	1.972	1.971	2.005
	SKEWNESS	0.033	−0.272	−0.298	−0.252	−0.186	−0.206
	KURTOSIS	0.819	0.686	0.695	0.627	0.618	0.612
Folk and Ward Method (φ)	MEAN	2.021	3.124	3.069	3.146	3.185	3.139
	SORTING	0.395	0.974	1.005	0.980	0.979	1.004
	SKEWNESS	−0.033	0.272	0.298	0.252	0.186	0.206
	KURTOSIS	0.819	0.686	0.695	0.627	0.618	0.612

**Table 6:** Corundum grains distribution statistics.

Concentration (%)		Fresh	15	17.5	20	22.5	25
Folk and Ward Method (μm)	MEAN	194.8	92.82	90.33	89.76	90.83	95.77
	SORTING	1.163	1.741	1.730	1.720	1.721	1.746
	SKEWNESS	−0.067	−0.162	−0.109	−0.094	−0.155	−0.261
	KURTOSIS	1.004	0.573	0.577	0.570	0.579	0.575
Folk and Ward Method (φ)	MEAN	2.360	3.429	3.469	3.478	3.461	3.384
	SORTING	0.218	0.800	0.791	0.782	0.783	0.804
	SKEWNESS	0.067	0.162	0.109	0.094	0.155	0.261
	KURTOSIS	1.004	0.573	0.577	0.570	0.579	0.575

**Table 7:** Olivine grains distribution statistics.

Concentration (%)		Fresh	15	17.5	20	22.5	25
Folk and Ward Method (μm)	MEAN	235.7	94.99	101.5	101.1	98.23	97.28
	SORTING	1.274	1.802	1.856	1.869	1.853	1.848
	SKEWNESS	−0.137	−0.104	−0.185	−0.169	−0.125	−0.102
	KURTOSIS	1.020	0.619	0.618	0.614	0.616	0.607
Folk and Ward Method (φ)	MEAN	2.085	3.396	3.300	3.306	3.348	3.362
	SORTING	0.349	0.850	0.892	0.902	0.889	0.886
	SKEWNESS	0.137	0.104	0.185	0.169	0.125	0.102
	KURTOSIS	1.020	0.619	0.618	0.614	0.616	0.607

**Table 8:** Recycled abrasives grains distribution statistics.

Abrasive Material		Alluvial Garnet	Recycled Garnet	Corundum	Olivine
Folk and Ward Method (μm)	MEAN	187.1	209.9	160.7	175.7
	SORTING	1.287	1.355	1.165	1.267
	SKEWNESS	0.071	0.098	0.029	0.181
	KURTOSIS	0.889	0.857	0.928	0.918
Folk and Ward Method (φ)	MEAN	2.418	2.252	2.638	2.509
	SORTING	0.364	0.438	0.221	0.341
	SKEWNESS	−0.071	−0.098	−0.029	−0.181
	KURTOSIS	0.889	0.857	0.928	0.918

## Data Availability

The data presented in this study are available on request from the corresponding author.
